# The Mechanisms Mediated by α7 Acetylcholine Nicotinic Receptors May Contribute to Peripheral Nerve Regeneration

**DOI:** 10.3390/molecules26247668

**Published:** 2021-12-18

**Authors:** Michael Sebastian Salazar Intriago, Roberta Piovesana, Alessandro Matera, Marilena Taggi, Rita Canipari, Cinzia Fabrizi, Claudio Papotto, Carlo Matera, Marco De Amici, Clelia Dallanoce, Ada Maria Tata

**Affiliations:** 1 Department of Biology and Biotechnology Charles Darwin, Sapienza University of Rome, 00185 Rome, Italy; michaelsebastian.salazarintriago@uniroma1.it (M.S.S.I.); roberta.piovesana@umontreal.ca (R.P.); alessandro.matera94@gmail.com (A.M.); 2 Department of Anatomy, Histology, Forensic Medicine and Orthopedics, Sapienza University of Rome, 00161 Rome, Italy; marilena.taggi@uniroma1.it (M.T.); rita.canipari@uniroma1.it (R.C.); cinzia.fabrizi@uniroma1.it (C.F.); 3 Department of Pharmaceutical Sciences, University of Milan, Via L. Mangiagalli 25, 20133 Milan, Italy; claudio.papotto@unimi.it (C.P.); carlo.matera@unimi.it (C.M.); marco.deamici@unimi.it (M.D.A.); 4 Research Center of Neurobiology (CRIN), Sapienza, University of Rome, 00185 Rome, Italy

**Keywords:** Schwann cells, α7 nicotinic acetylcholine receptor, ICH3, inflammation, regeneration, IL-6, metalloproteinases

## Abstract

Due to the microenvironment created by Schwann cell (SC) activity, peripheral nerve fibers are able to regenerate. Inflammation is the first response to nerve damage and the removal of cellular and myelin debris is essential in preventing the persistence of the local inflammation that may negatively affect nerve regeneration. Acetylcholine (ACh) is one of the neurotransmitters involved in the modulation of inflammation through the activity of its receptors, belonging to both the muscarinic and nicotinic classes. In this report, we evaluated the expression of α7 nicotinic acetylcholine receptors (nAChRs) in rat sciatic nerve, particularly in SCs, after peripheral nerve injury. α7 nAChRs are absent in sciatic nerve immediately after dissection, but their expression is significantly enhanced in SCs after 24 h in cultured sciatic nerve segments or in the presence of the proinflammatory neuropeptide Bradykinin (BK). Moreover, we found that activation of α7 nAChRs with the selective partial agonist ICH3 causes a decreased expression of c-Jun and an upregulation of uPA, MMP2 and MMP9 activity. In addition, ICH3 treatment inhibits IL-6 transcript level expression as well as the cytokine release. These results suggest that ACh, probably released from regenerating axons or by SC themselves, may actively promote through α7 nAChRs activation an anti-inflammatory microenvironment that contributes to better improving the peripheral nerve regeneration.

## 1. Introduction

During nerve repair, Schwann cells (SCs) and macrophages play essential roles in tissue homeostasis, promoting nerve regeneration [1,2,3,4]. Previous studies demonstrated that different neurotransmitters may control and modulate a variety of biological processes during development as well as adult life physiology [5,6,7]. In particular, it has been shown that both rat and human SCs express muscarinic acetylcholine receptors (mAChRs) and are thus able to respond to acetylcholine (ACh) stimuli [8,9,10,11]. The selective activation of specific mAChRs, such as the M2 subtype, regulates some aspects of the SCs development [8,9,10,11] as well as those mechanisms aimed at enhancing their regenerative capability, modulating the production and release of the nerve growth factor (NGF) [12]. Therefore, the cholinergic system could be a putative target for identifying novel pharmacological treatments to speed up the regeneration process. Worth noting, in several different districts, the cholinergic signal is also mediated by activation of the composite family of nicotinic acetylcholine receptors (nAChRs). Furthermore, a remarkable correlation between the cholinergic system and the immune system modulation has recently emerged [13]. Based on the anti-inflammatory properties associated to the α7 nAChR [14,15], we investigated a potential contribution of this receptor subtype to nerve repair following peripheral injury. To the best of our knowledge, the role of nAChRs in SCs is unknown, and we initially evaluated the ex vivo expression of the α7 nAChR in sciatic nerves, evidencing its increased expression in SCs after sciatic nerve axotomy and during the inflammatory phase. To characterize a potential anti-inflammatory effect of the α7 nAChR activation after peripheral nerve injury, we treated cultured ex vivo sciatic nerve segments and primary SCs with a selective activator of this receptor subtype. 

In the last decade, some of us have been active in the study and characterization of α7 nAChRs and their therapeutic potential [16,17,18,19,20,21,22,23,24,25,26]. In this framework, we designed, synthesized and tested (*R*)-(-)-3-methoxy-1-oxa-2,7-diaza-7,10-ethanospiro[4.5]dec-2-ene sesquifumarate (from now on identified as ICH3), which behaves as a selective orthosteric partial agonist of the α7 receptor subtype [19]. ICH3, whose pharmacological profile was previously investigated *in vitro* [27] and in vivo [28,29,30], was thus selected as a model molecular probe in this new study.

Our initial data showed that α7 nAChRs are specifically expressed in Schwann cells after sciatic nerve dissection, suggesting a role for this receptor after peripheral nerve injury. In this respect, we found that selective activation of α7 nAChRs by ICH3 reduced the IL6 production and increased the metalloproteinase activity, promoting a microenvironment beneficial to peripheral nerve regeneration.

## 2. Results

### 2.1. Expression of α7 Nicotinic Receptor in Schwann Cells after Sciatic Nerve Dissection

**Figure 1:**
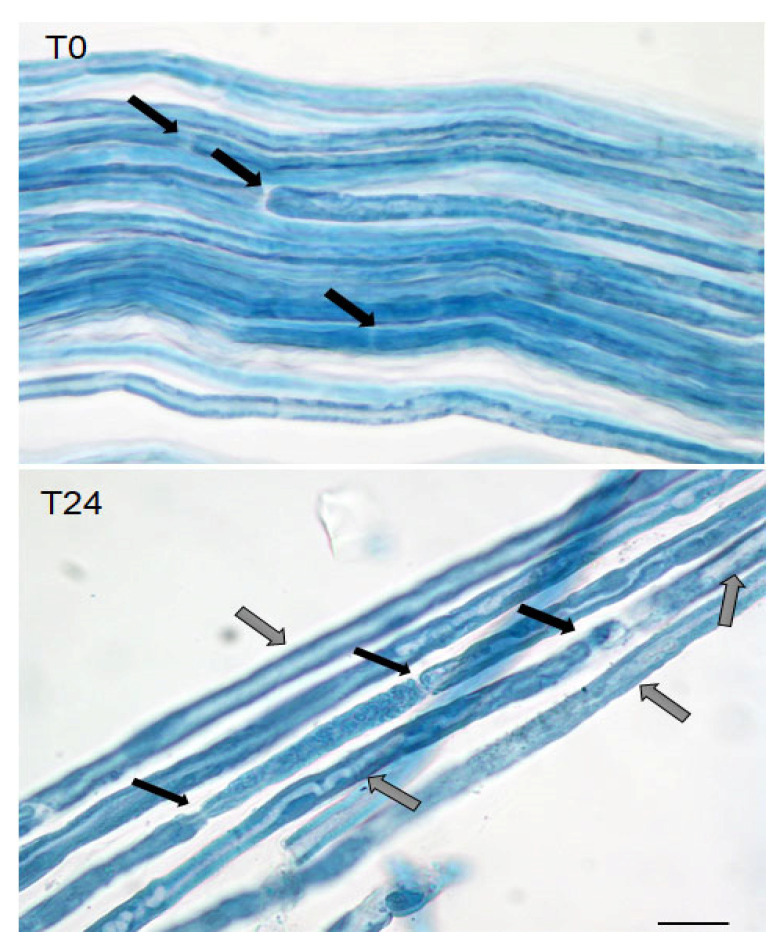
Sudan black staining of teased fibers from sciatic nerves. The fibers were analyzed immediately after dissection (T0) or after 24 h (T24) in culture. Black arrows indicate Ranvier nodes. The internode regions present a good levels of myelination at T0 with rare areas of demyelination (white areas). After 24 h from dissection, the areas of demyelination (white areas) are more evident (grey arrows) (scale bar = 20 µm).

In our previous studies, we demonstrated that SCs are cholinoceptive since they express mAChRs and respond to muscarinic mimetics modulating SCs proliferation and differentiation [8,12]. To detect the expression of the α7 nicotinic receptor, sciatic nerves from adult Wistar rats were dissected and fixed. The teased fibers were firstly observed after Sudan black staining to evaluate the myelin conservation in the fibers immediately after dissection (T0) or after 24 h in culture (T24). As can be observed in Figure 1, the teased fibers after dissection present a good myelin organization with rare areas of demyelination (white areas) that may be also considered Schmidt Lateran incisures. After 24 h from dissection, large areas of demyelination are visible.

**Figure 2:**
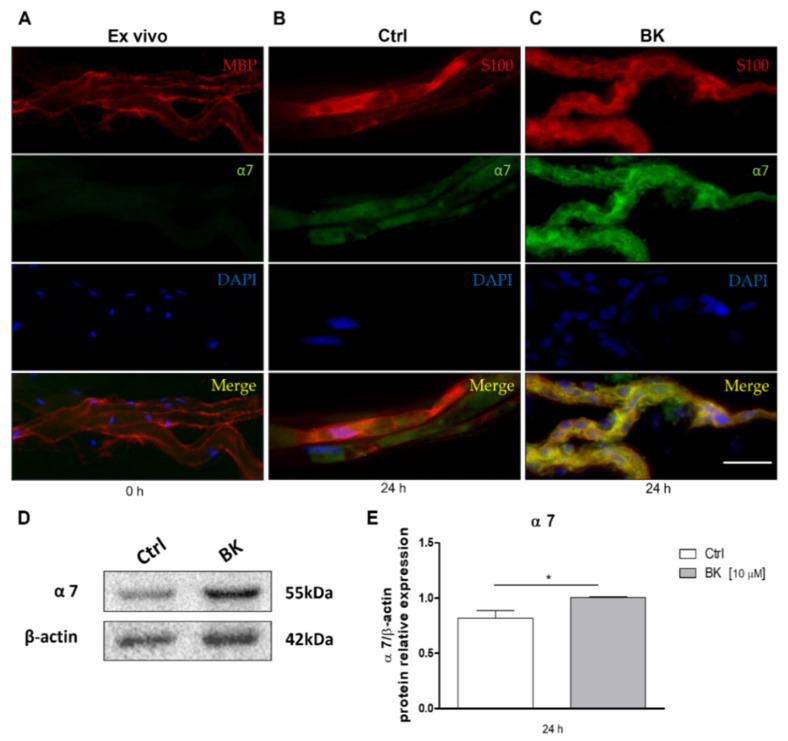
α7 nAChRs expression in sciatic nerve after axotomy and in cultured Schwann cells. (**A**–**C**) Immunostaining for α7 nAChRs (green) and MBP or S100 β (red) was performed on teased fibers from sciatic nerve ex-vivo (**A**) or in cultured nerve segments maintained for 24 h in the absence (**B**) or presence (**C**) of 10 µM BK; scale bar: 50 µm. (**D**) The α7 nAChRs expression was also evaluated by Western blot analysis on cultured Schwann cells in the absence or presence of 10 µM BK. (**E**) Graph shows the densitometric analysis of the bands obtained from three independent experiments, normalized against the housekeeping protein, GAPDH (n = 3; * *p* < 0.05).

The teased fibers in these experimental conditions were stained for the α7 nAChR and for the myelin basic protein (MBP), to assess a possible colocalization of the two proteins. As reported in Figure 2A, immediately after dissection the sciatic nerve did not show any immunopositivity for the α7 nAChR, whereas the MBP protein appeared distributed peripherally, indicating the organization of myelin lamellae around the fibers. Since it has been already ascertained in various systems that the α7 nAChR is involved in anti-inflammatory processes [15,31], we repeated the experiment maintaining sciatic nerves in culture for 24 h after axotomy, with or without an additional inflammatory stimulus that was provided by the neuropeptide Bradykinin (BK). As shown in Figure 2B,C, α7 nAChR protein levels were found to be significantly increased already after 24 h after dissection, but its expression was further enhanced when 10 μM BK was added to the culture medium. The α7 nAChR immunostaining colocalizes with the SCs marker S100β, demonstrating that this receptor subtype is expressed by the SCs after peripheral nerve axotomy.

In order to validate this result, we analyzed the expression of α7 nAChR in cultured Schwann cells maintained in the presence or absence of the inflammatory stimulus mediated by BK. The Western blot analysis clearly indicated that α7 receptors were faintly expressed in SCs under basal conditions, but their expression significantly increased upon 10 µM BK treatment (Figure 2D,E).

### 2.2. Analysis of the Expression of c-Jun Transcription Factor in Sciatic Nerves

**Figure 3:**
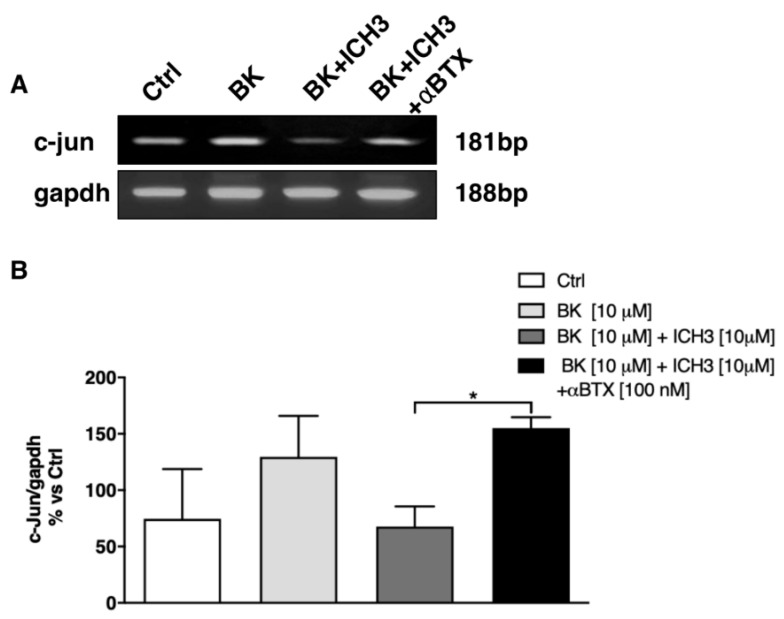
c-Jun transcript level expression in cultured sciatic nerve after BK and ICH3 treatment. Sciatic nerves were placed in media containing the inflammatory agent BK for 24 h followed by an additional 24 h in the presence of the α7 agonist ICH3 in the absence or presence of α7 antagonist αBTX. (**A**) RT-PCR analysis of c-Jun expression; GAPDH was used as the housekeeping gene. (**B**) Graph shows the densitometric analysis of the bands obtained from three independent experiments, normalized against the housekeeping gene. (n = 3; * *p* < 0.05).

The transcriptional factor c-Jun is known to play a key role in nerve regeneration, because it is responsible for the SCs dedifferentiation and the acquisition of the repair SCs phenotype (see below), relevant to restoring SCs proliferation and to neurotrophic factor production [2,32]. By semiquantitative RT-PCR experiments, we analyzed the expression of c-Jun transcript levels in rat sciatic nerves maintained in culture for 24 h after axotomy, in different conditions according to the experimental protocol. As detailed in Figure 3A, c-Jun transcripts are already present in the sciatic nerve after 24 h from dissection (Ctrl) and their expression increases following 24 h exposure to the BK (10 μM) treatment. Interestingly, activation of the α7 nAChR, using the selective agonist ICH3 (10 μM) [19] in the presence of the inflammatory peptide BK (10 μM), caused a decrease in c-Jun expression. To confirm that this effect is mediated by the selective activation of the α7 nAChR, sciatic nerves were pretreated with the α7 nAChR antagonist α-Bungarotoxin (α-BTX, 100 nM) before ICH3 treatment. In the presence of α-BTX, the expression level of c-Jun was found to be comparable to that detected upon BK treatment (Figure 3B).

### 2.3. Analysis of the Expression and Release of IL-6

**Figure 4:**
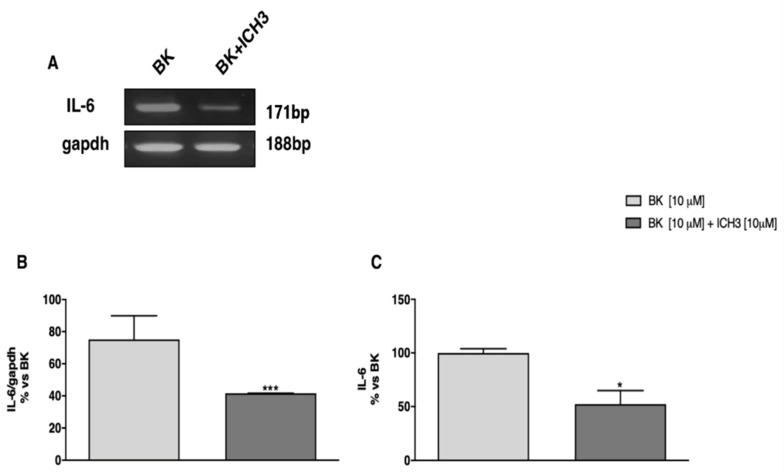
IL-6 expression and release upon α7 nAChR agonist treatment. (**A**) RT-PCR analysis indicates a decrease of IL-6 transcript expression after ICH3 treatment. (**B**) Graph shows the densitometric analysis of the bands obtained from three independent experiments, normalized against the housekeeping gene, GAPDH. (n = 3; *** *p* < 0.001). (**C**) ELISA assay was performed on culture media of ex vivo sciatic nerves, maintained for 24 h in different experimental conditions. A significant decrease of IL-6 releases after 24 h of ICH3 exposure is detected. The analysis was performed calculating the percentage of IL-6 released/well and compared to the average of samples containing BK alone (n = 3; * *p* < 0.05).

Using semiquantitative RT-PCR experiments, we found that activation of the α7 nAChR by ICH3 negatively regulated the expression of the proinflammatory cytokine IL-6 in cultured sciatic nerves. As reported in Figure 4A,B, sciatic nerves treated for 24 h with ICH3 (10 μM) and the inflammatory peptide BK (10 μM) showed a significant reduction in transcriptional levels of IL-6 compared to the condition in which BK was present alone. Moreover, in view of the observed significant transcriptional modulation, we analyzed the levels of IL-6 released in the culture media of the sciatic nerves maintained in different experimental conditions. As shown in Figure 4C, by using the enzyme-linked immunosorbent assay (ELISA), we observed that the levels of IL-6 released in the medium decreased significantly following the activation of the α7 nAChR by ICH3 (10 μM) and the BK (10 μM) treatment, compared to the condition in which only BK was present.

### 2.4. Analysis of Metalloproteinase Activity in Sciatic Nerve Culture Media

**Figure 5:**
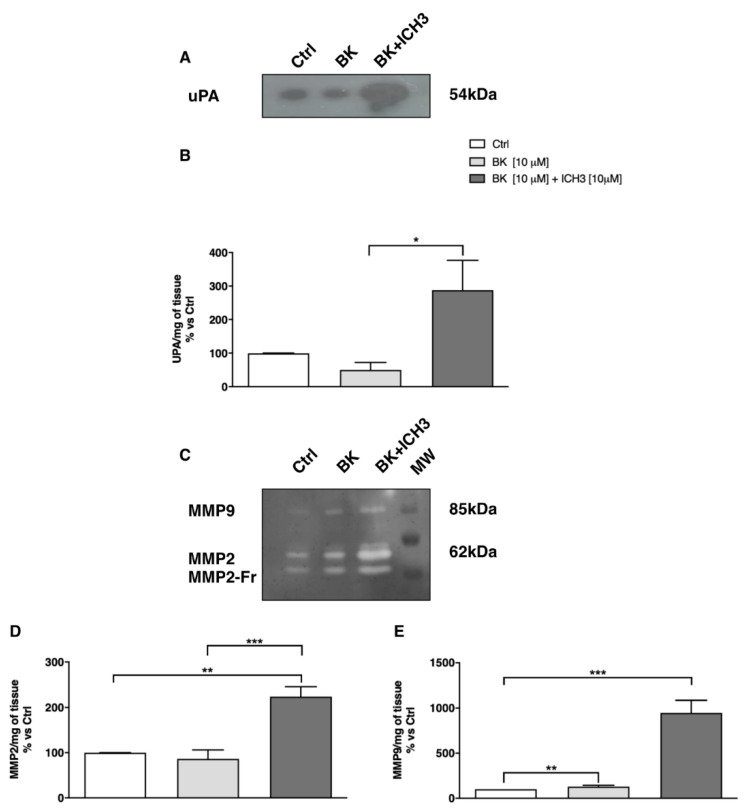
uPA and MMP2/MMP9 activity measured in the media of cultured sciatic nerves by zymography. (**A**,**B**) uPA activity is significantly increased after ICH-3 treatment (n = 3; * *p* < 0.05). (**C**,**D**) MMP2 and (**C**–**E**) MMP9 activities are significantly increased after 24 h of ICH-3 treatment (n = 3; ** *p* < 0.01; *** *p* < 0.001). PA and MMPs activities were expressed as percentage of control arbitrarily set at 100, and values were normalized to the value in milligrams of nerve tissue present in the culture dish.

It is known that metalloproteinases are essential during the regenerative processes in the peripheral nervous system, since their action promotes matrix remodeling and maturation of neurotrophic factors, necessary to neuronal survival and axonal regeneration. By means of zymography, we analyzed the activity of metalloproteinases 2 and 9 (MMP2 and MMP9, respectively) and of the plasminogen activator urokinase (uPA) in sciatic nerve culture media. As reported in Figure 5A,B, we observed a significant increase in the uPA activity following activation of the α7 nAChR by ICH3. Similarly, we found a significantly higher activity of MMP2 and MMP9 after ICH3 (10 μM) and BK (10 μM) treatment, compared to the samples exposed to BK alone (Figure 5C–E).

## 3. Discussion

The peripheral nervous system is characterized by a high regenerative capability, largely depending on SC plasticity. Following traumatic injuries, peripheral nerves undergo a multistep repair program including Wallerian degeneration, axonal regrowth, target reinnervation and axon remyelination. During this program, SCs change their phenotype into a repair Schwann cells phenotype, which promotes axon elongation and drives the proximal stump toward the previously innervated target organ [2,3,32]. In addition, these *Repair Schwann Cells*, together with macrophages, trigger the inflammatory response to axonal damage [33]. The balance between the inflammatory and regenerative responses is critical for a proper nerve regeneration, which is considered to have been accomplished when the inflammatory process is resolved and the repair Schwann cells assume again a myelinating or non-myelinating phenotype, according to the caliber of the axon [34].

In these last years, it has been demonstrated that, among the various biological functions exerted by ACh, this neurotransmitter is involved also in morphogenetic actions through the regulation of some stages of neuron and glial cell differentiation [8,9,10,35,36]. Several studies were conducted in the peripheral nervous system where the cholinergic receptors have been identified in SCs, revealing that glial cells are able to respond to cholinergic stimuli [11,12,37]. Interestingly, ACh is present in sciatic nerves and can be potentially released in extrasynaptic regions both by motor and sensory axons [38], suggesting its involvement in axon-glia crosstalk. However, when isolated by the axons and cultured in vitro, SCs are able to synthesize and release ACh that may be useful to contribute to the balance between the proliferative and differentiative phase [39].

In our previous investigations, we also proved that rat and human SCs express the various mAChR subtypes (M1, M2, M3, M4) [8,11] and characterized in detail the effects downstream of the M2 mAChR activation. Indeed, stimulation of the latter receptor subtype induces a reversible block of SC proliferation as well as a reduction of the expression levels of transcription factors involved in the maintenance of the undifferentiated state, thus promoting the acquisition of a myelinating phenotype [9,10]. In the present study, we focused on the role of ACh in the process of peripheral nerve regeneration through the activation of nicotinic receptors. Among the several nAChRs, accumulating evidence indicates that the α7 subtype modulates the so-called “cholinergic anti-inflammatory pathway” both in the immune systems and in the brain [15,40,41,42]. Our initial results showed that ex vivo sciatic nerves weakly express the α7 nAChR. Conversely, its expression significantly increased in sciatic nerve explants and then in cultured sciatic nerve segments treated with the proinflammatory peptide BK. The expression of α7 nAChR observed on sciatic nerves is located on SCs as demonstrated by its coexpression with SC markers such as MBP or S100β. Considering the key role exerted by SCs in the regeneration process and the significant increase of α7 nAChR expression after injury, our data suggest a peculiar role of this receptor subtype when SCs are involved in the regenerative events. This hypothesis is corroborated by the results obtained after selective stimulation of the α7 nAChR with the agonist ICH3, previously characterized as an orthosteric selective activator of the α7 nAChR subtype [19]. We observed that stimulation of α7 nAChRs counteracted the BK-induced effects. In fact, BK induced an increased expression of the c-Jun transcript, a typical marker of the repair SCs phenotype, and this increase was counterbalanced by selective activation of the α7 nAChR with ICH3. Pretreatment of sciatic nerve cultures with the selective α7 nAChR antagonist α-Bungarotoxin (α-BTX) strongly hindered the effect of ICH3, causing an expression of the c-Jun transcripts comparable to that observed in the presence of BK. Therefore, these results suggest a specific role of the α7 nAChR in the modulation of SCs plasticity, particularly in their reacquisition of a mature phenotype. Based on the role of α7 nAChR in mediating anti-inflammatory processes [15], we also evaluated the effects of ICH3 stimulation on the production and release of IL-6 proinflammatory cytokine. As expected, the cultured sciatic nerve expresses and releases IL-6 in the culture media after the fiber axotomy and BK stimulation. Interestingly, the α7 nAChR stimulation caused a significant reduction of IL-6 transcript levels and release, confirming its role in restoring tissue homeostasis. It also enhanced the activity of the matrix metalloproteinases 2 and 9 (MMP2 and MMP9) as well as the plasminogen activator urokinase (uPA) in sciatic nerve culture media. The action of these proteases is essential to the process of regeneration in the peripheral nervous system, since they promote remodeling of the extracellular matrix stimulating SC migration and axon elongation. Moreover, the action of MMP2 is relevant to the maturation of the nerve growth factor (NGF), produced by SCs and necessary to support neuron survival and axonal regeneration [43,44]. Taken together, these results suggest that in sciatic nerves after peripheral nerve injury, ACh, probably released by regenerating axons or directly produced by SCs, may appreciably contribute to engender a microenvironment beneficial for nerve regeneration using the α7 nAChRs activation, specifically expressed in SCs after axon damage. However, considering that macrophages [45,46] and fibroblasts [47] express α7 nAChRs, we can hypothesize that all cell populations localized in the sciatic nerve may contribute to the recovery of the tissue homeostasis necessary to stabilize nerve regeneration.

## 4. Materials and Methods

### 4.1. Animals

All procedures involving animals were carried out in accordance with the guidelines of the European Communities Council Directive (86/609/EEC of 24 November 1986) and the Italian National Law DL/116/92. All methods were in accordance with guidelines of the protocols n. 7FF2C.6.EXT.96 that was approved by the Ministry of Health (AMT, Aut. N. 1184/2016-PR 16/12/2016). All animals were housed in a temperature-controlled room (22 ± 1 °C) with a 12 h light/dark cycle and free access to food and water.

### 4.2. Ex Vivo Sciatic Nerve Explants

Sciatic nerves were harvested from adult male Wistar rats. Each nerve was cut in 2 pieces of about 3 cm segments (35 mg/each). The nerves were cultured for 24 h in Dulbecco′s modified Eagle′s medium (DMEM, Sigma-Aldrich, St. Louis, MO, USA) supplemented with 0.5% (*v/v*) fetal bovine serum (FBS, Sigma-Aldrich, St. Louis, MO, USA) and 2 μM Forskolin (Fsk, Sigma-Aldrich, St. Louis, MO, USA). For immunohistochemistry experiments, nerves were fixed with 4% paraformaldehyde (PFA, Sigma-Aldrich, St. Louis, MO, USA) in PBS overnight at 4 °C and then the fibers were teased on positively charged glass slides.

### 4.3. Schwann Cell Cultures

Schwann cells (SCs) were obtained from sciatic nerves dissected from 2-day-old Wistar pups, according to the protocol described by Brockes [48] and modified by Davis and Stroobant [49]. In brief, sciatic nerves were digested with trypsin/collagenase (Type I, Sigma-Aldrich, St. Louis, MO, USA) and seeded into 25 cm^2^ cell culture flasks with fresh Dulbecco’s modified Eagle’s medium (DMEM, Sigma-Aldrich, St. Louis, MO, USA) containing 10% (*v/v*) fetal bovine serum (FBS, Sigma-Aldrich, St. Louis, MO, USA). To selectively remove fibroblasts, cells were treated with 1 mM cytosine arabinoside (AraC, Sigma-Aldrich, St. Louis, MO, USA) for 48 h and then with anti-Thy 1.1 (1:1000, Serotec, Bio-Rad group, Hercules, CA, USA) and rabbit complement (1:2 *v/v*; Cedarlane, Burlington, ON, Canada). SCs were then amplified in DMEM supplemented with 10% (*v/v*) fetal bovine serum (FBS, Sigma-Aldrich, St. Louis, MO, USA), 1% (*v/v*) Penicillin/ Streptomycin (Sigma-Aldrich, St. Louis, MO, USA), 1% (*v/v*) Glutamine (Sigma-Aldrich, St. Louis, MO, USA), 5µM forskolin (Fsk, Sigma-Aldrich, St. Louis, MO, USA) and bovine pituitary extract (1:150, Sigma-Aldrich, St. Louis, MO, USA) [9,10]. Purified Schwann cells were cultured on 0.01% (*v/v*) poly-l-lysine precoated dishes (Sigma-Aldrich, St. Louis, MO, USA) in DMEM, 10% (*v/v*) FBS, 2 μM Fsk and 10 ng/mL neuregulin-1 (NRG1, Immunological Sciences, Rome, Italy) during subsequent experiments [11,12]. The cultures were maintained at subconfluent levels in a 37 °C in a humidified 10% CO_2_ atmosphere.

### 4.4. Pharmacological Treatments

(*R*)-(-)-3-Methoxy-1-oxa-2,7-diaza-7,10-ethanospiro[4.5]dec-2-ene sesquifumarate (ICH3), the selective partial agonist for the α7 nAChR, was synthesized according to a published procedure [19], and was utilized at the final concentration of 10 μM. The α7 nAChR α-Bungarotoxin (Tocris Bioscience, Bristol, UK) was used at a final concentration of 100 nM and was added 2 h before the treatment with ICH3. The proinflammatory peptide Bradykinin (BK, Sigma-Aldrich, St. Louis, MO, USA) was used at the final concentration of 10 μM and in general was added 24 h before the ICH3 treatment to favor the increase of α7 nAChR expression. Controls were obtained maintaining the cells in the growth medium alone.

### 4.5. RNA Extraction and RT-PCR Analysis

Total RNA was extracted from sciatic nerves using Tri-Reagent (Sigma-Aldrich, St. Louis, MO, USA). RNA concentration and purity were detected using the NanoDrop Lite Spectrophotometer (Thermo, Dreieich, Germany). One microgram of total RNA was reverse-transcribed into cDNA for 60 min at 37 °C with 1 μg of random primers (Promega, Milan, Italy) and 200U of Moloney Murine Leukemia Virus (M-MLV) reverse transcriptase (Promega, Milan, Italy). Glyceraldehyde-3-phospate dehydrogenase (GAPDH) was used as a housekeeping gene. The densitometric analysis of the RT-PCR bands was performed using the ImageJ imaging software (NIH, Bethesda, MD, USA).

The sequences of the primers used were:

*c-Jun:* Forward 5′-GCGCGCCCTAGCTGAACTGC-3′
Reverse 5′-AGTTGCTGAGGTTGGCGTAG-3′
*Il-6:* Forward 5′-TGGTCTTCTGGAGTTCCGTT-3′
Reverse: 5′-AGAGCATTGGAAGTTGGGGT -3′
*GAPDH:* Forward 5′-TGGCATTGTGGAAGGGCTCATGAC-3′
Reverse 5′-ATGCCAGTGAGCTTCCCGTTCAGC-3′

### 4.6. Western Blot

Cells were collected using trypsin and centrifuged for 10 min at 1000 rpm. The whole-cells lysates were extracted in lysis buffer (10 mM TrisEDTA, 0.5% NP40, 150 mM NaCl) supplemented with the protease inhibitors cocktail (Sigma-Aldrich, St. Louis, MO, USA). After 20 min of incubation on ice, samples were sonicated for 15 s and then centrifuged for 10 min at 14,000 rpm at 4 °C. Protein concentration was determined using the BCA Protein Assay Kit (Thermo Scientific, Waltham, MA, USA), according to the manufacturer’s protocol. Sample buffer (4×) was added to the protein sample and boiled for 5 min. The protein extracts (50 μg for sample) were run on 10% SDS-polyacrylamide gel (SDS-PAGE) at 100 V using running buffer (0.25 M Tris, 2.4 M Glycine, 0.035 M SDS) and transferred to a polyvinylidene difluoride (PVDF) membrane (Merck Millipore, Darmstadt, Germany) at 80 V in transfer buffer (20 mM Tris; 150 mM glycine, 10% [*v/v*] methanol) for 1 h. Membranes were blocked for 1 h in 5% of nonfat milk powder (Sigma-Aldrich, St. Louis, MO, USA) in Tris-buffered saline (TBS) containing 10% Tween-20, and then incubated with the antibodies overnight at 4 °C. Primary antibodies used were: rabbit anti-α7 antibody (1:500, Bioss, MA, USA, bs-1049R) and mouse anti-β-actin (1:2000; Immunological Sciences, Rome, Italy; AB-24008). After overnight incubation, membranes were washed three times with TBS containing 0.1% Tween-20 and then incubated for 1 h at room temperature (RT) with a secondary antibody: antirabbit horseradish peroxidase (1:10,000, Promega, Italy) or antimouse horseradish peroxidase (1:10,000, Immunological Sciences, Rome, Italy). Membranes were exposed to the ECL chemiluminescence reagent (Immunological Science, Rome, Italy) for signal detection. The bands were detected by exposition to Chemidoc (Molecular Imager ChemiDoc XRS + System with Image Lab Software, Bio-Rad, CA, USA). Band intensities were quantified as optical density using the ImageJ imaging software (NIH, Bethesda, MD, USA).

### 4.7. Enzyme-Linked Immunosorbent Assay (ELISA)

Media were collected from sciatic nerves cultures maintained for 24 h in different conditions according to the experimental plan. The levels of interleukin-6 (IL-6) were determined in cell media by using a two-site immunoenzymatic assay (Fine Test, Wuhan, Hubei, China). The procedures were performed in accordance with the manufacturer’s instructions. The protein detection of the samples was previously evaluated. Each point of the standard curve and samples were measured in duplicate. The colorimetric reaction was measured in absorbance mode at 450 nm by a Multiskan GO spectrophotometer (Thermo Scientific, Rodano, Milan, Italy).

### 4.8. Zymography

Gelatinolytic activity of conditioned media was assayed as previously described [12]. The enzymatic activity of MMP2 and MMP9 was determined by gelatin zymography. Briefly, media of sciatic nerve in culture were analyzed on 7% sodium dodecyl sulfate polyacrylamide gel electrophoresis SDS-PAGE, containing 0.1% gelatin under nonreducing conditions. After electrophoresis, gels were washed twice with distilled water containing 2.5% Triton-X100 for 30 min at RT to remove SDS, and then incubated in collagenase buffer (0.5 M Tris-HCl pH7.5, 50 mM CaCl2 and 2 M NaCl) overnight at 37 °C. The gels were stained with Coomassie brilliant blue R-250 and washed with distaining solution (30% methanol, 10% acetic acid, and 60% water). To test the enzymatic activity of uPA, aliquots of the sciatic nerve media were separated by electrophoresis in 10% SDS-PAGE under nonreducing conditions, according to the procedure of Laemmli [50]. The uPA was then visualized by placing the Triton-X100-washed gel on a casein–agar–plasminogen underlay. The lytic zones were plasminogen dependent. The gels were photographed and the densitometric analysis was performed using the ImageJ software (National Institutes of Health, NIH, 469 Bethesda, MD, USA) to obtain a semiquantitative estimation of protease activities. Molecular weights were calculated from the position of pre-stained markers subjected to electrophoresis in parallel lines. Densitometric scanning of zymographies was performed to derive a semiquantitative estimation of protease activities. The analysis was performed on five independent experiments.

### 4.9. Teasing, Hystological Analysis, and Immunostaining of Sciatic Nerves

Sciatic nerves were collected, washed three times with PBS 1X. Cultured nerves were maintained in culture with DMEM supplemented with 10% FBS, 2 μM Forskolin and a different pharmacological treatment according to the experimental plan. Tissues were then fixed with paraformaldehyde (PFA) 4% for 24 h and teased on superfrost slides (Thermo Scientific, Rodano, Milan, Italy). Firstly, connective tissue was removed to allow the separation of the single fibers. Teased nerves were left to dry for 24 h at RT.

For Sudan Black B staining used for histological analysis, teased fibers were immersed in 0.1% Sudan black B diluted in 70% ethanol for 20 min at RT. Slides were then washed three times for 5 min each in PBS containing 0.02% Tween 20. After a final wash with PBS, the fibers were overlaid with coverslips using Vectashield mounting solution and imaged with Nikon light microscope (Eclipse E600; Nikon Instruments SpA) [51].

For the immunostaining, the samples were blocked with PBS 1X + 1% bovine serum albumin (BSA) + 10% normal goat serum at RT for 45 min. For α7 nAChR and S100β staining, 0.1% Triton X-100 was added to the blocking solution. Incubation of the primary antibody [rabbit anti-α7 [Bioss Antibodies 1:100 (*v/v*)]; mouse anti-MBP, [Abcam 1:100 (*v/v*)]; mouse anti-S100β, [Sigma 1:500 (*v/v*)] was performed in the blocking solution at 4 °C overnight. Three washes with PBS 1X were performed to remove unbound primary antibody. Secondary antibodies, diluted in blocking solution, were then incubated for 3 h at RT; goat antirabbit IgG, Alexa Fluor 488.conjugated antibody [Immunological Sciences, Rome, Italy, 1:100 (*v/v*)] and goat antimouse IgG, Alexa-Fluor 594 conjugated antibody [Immunological Sciences, Rome, Italy, 1:100 (*v/v*)]. After other three washes in PBS 1X, the samples were mounted with antifade mounting medium with 4′,6-diamidino-2-phenylindole (DAPI, Immunological Science, Rome, Italy). Images were obtained by a fluorescence microscope Zeiss ApoTome (Axio Imager Z1 Stand, Oberkochen, Germany).

### 4.10. Statistical Analysis

Student’s t test and one-way ANOVA test, followed by Bonferroni’s post hoc test, were used to evaluate statistical significance within the different samples. The results were considered statistically significant at *p* < 0.05 (*), *p* < 0.01 (**) and *p* < 0.001 (***).

## 5. Conclusions

The present data reinforce previous results obtained by our research group, reaffirming that acetylcholine, probably released in extra-synaptic regions of motor and sensory axons [38], may significantly contribute to Schwann cell development and plasticity through the activation of different cholinergic receptor types. In this study, we assessed the expression of the α7 nAChR in Schwann cells after peripheral nerve injury and proved that the selective activation of this receptor subtype may be relevant to the establishment of the microenvironment favorable to improving nerve regeneration. In this case, the ACh necessary to stimulate the α7 nAChR may be directly produced by Schwann cells that acquire the ability to release ACh when isolated from the axons [39]. However, we cannot exclude that in vivo ACh may also derive from regenerating axons and the effects mediated by α7 nAChRs could contribute to switch off peripheral inflammation and to rescue the SC differentiated phenotype by downregulation of c-Jun expression.

Although further analyses should be performed, these data contribute to increase the knowledge of the molecular mechanisms operating after peripheral nerve injury and may provide new therapeutic tools aimed at promoting the nerve regeneration process. In this perspective, the identification of new compounds endowed with a selective agonistic profile for α7 nAChRs may be strategic to the clinical treatment of the traumatic peripheral nerve injuries.

## Data Availability

Not applicable.
